# Small Pox

**Published:** 1860-10

**Authors:** 


### SMALL POX.

FROM a very wide field of observations diligently made and carefully collated, European statisticians have arrived at the following conclusions:

Of one hundred persons vaccinated, and who subsequently take the small pox, six die; while of one hundred unvaccinated, who take the disease, six times that many die, or thirty-six out of every hundred; in other words, the vaccinated man, if he does take the small pox, has six chances of getting well, while the unvaccinated has only one.

Infantile vaccination has of late years become less efficient than formerly, not that there is less protecting power in vaccination, but because it is done too negligently, or because there has been remissness in procuring good vaccine matter from healthy sources; and it may be that the vaccine matter has deteriorated since its introduction by the immortal Jenner, three quarters of a century ago; therefore, one of two courses should be followed, either have the child re-vaccinated at the age of ten years by a careful physician, who would take the utmost pains to obtain good matter, or have a cow innoculated with the matter of small pox from a man; then, that which the cow produces will be fresh, pure, and powerful; this would give a new and unadulterated article, sufficient for a whole country for half a century to come.

The Prussian, more than any government in existence, practices vaccination, and every soldier is re-vaccinated on entering the army, which numbers several scores of thousands, the result being, that during 1859, there were only two deaths from small pox. Out of one hundred persons vaccinated in infancy, seventy "take" when re-vaccinated on entering the Prussian army. Varioloid is when small pox is taken after vaccination

